# Supplementary material for: Implications of the 2019–2020 megafires for the biogeography and conservation of Australian vegetation
Source: Nat Commun. 2021 Feb 15;12:1023. doi: 10.1038/s41467-021-21266-5 (PMC7884386; doi:10.1038/s41467-021-21266-5)
Supplement: Supplementary file 1 — Supplementary Information [file 41467_2021_21266_MOESM1_ESM.pdf]

Implications of the 2019-2020 Megafires for the Biogeography and Conservation of Australian Vegetation

Supplementary Information

Supplementary Figure 1. Rainfall and temperature across Australia prior to the 2019-2020 fire season.

Supplementary Figure 2. Progression of the 2019-2020 fires in eastern Australia between 1 July 2019 and 11 February 2020.

Supplementary Figure 3. Species richness of burnt and unburnt refugial populations of range restricted and moderately widespread taxa, non-rainforest, sclerophyll forest and woodland taxa, species found in rocky habitats, and epiphytes.

Supplementary Table 1. Estimated area (ha) and percent of total (%) of broad vegetation types burnt during the 2019-2020 fires within 11 south-east Australian IBRA bioregions

Supplementary Table 2. Number of vascular plant taxa in different life forms by rainforest habitat category.

Supplementary Table 3. Counts of species endemic to the study area classified by life form and range size class.

Supplementary Notes- consisting of:

Supplementary Notes 1: Further details of range extent (RE_T) by habitat type (rainforest) category analysis.

Supplementary Notes 2: Further details of quantile regression of proportion burnt (PF_M) by range extent (RE_T).

Supplementary Notes 3: Contingency analysis of life form by range extent (RE_T)

Supplementary Notes 4: List of R packages used

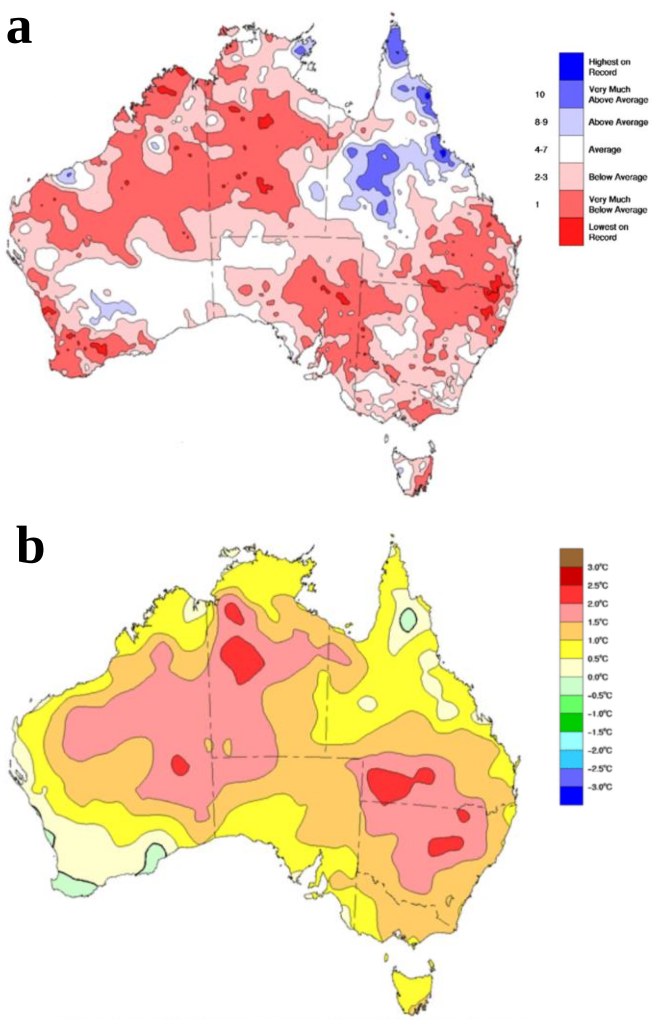

Supplementary Figure 1. Rainfall and temperature across Australia prior to the 2019-2020 fire season. (a) Rainfall deciles for the period 1 August 2018 – 31 July 2019, showing very low to record-low rainfall across much south-eastern Australia. (b) Mean temperature anomaly, 1 August 2018 – 31 July 2019. (a) and (b) were obtained from the Australian Government Bureau of Meteorology under Creative Commons Attribution 3.0 Australia (CC BY 3.0 AU) Licence.

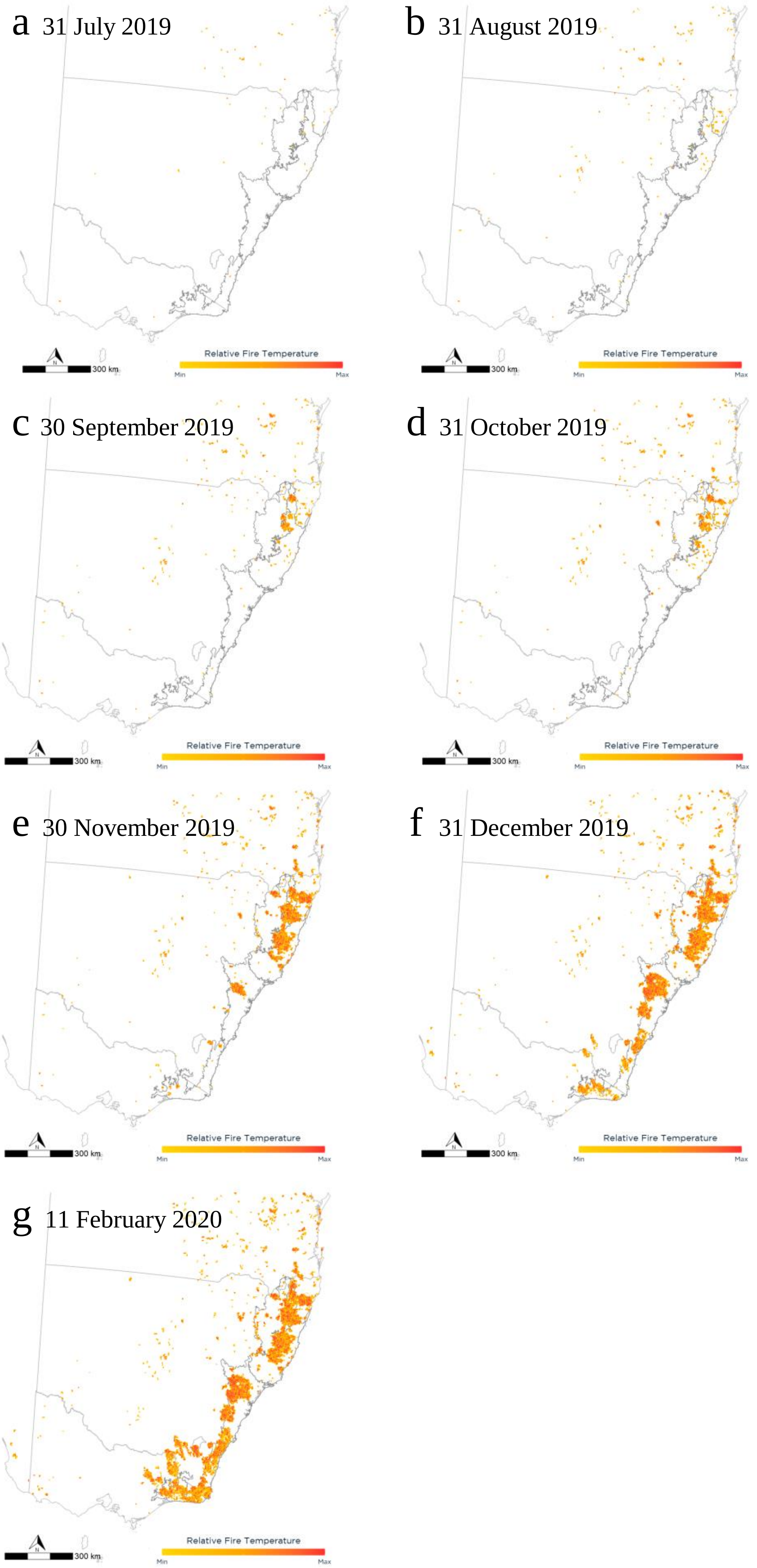

Supplementary Figure 2. Progression of the 2019-2020 fires between 1 July 2019 and 11 February 2020. (a-g) Cumulative extent of fires between 1 July 2019 and each date.

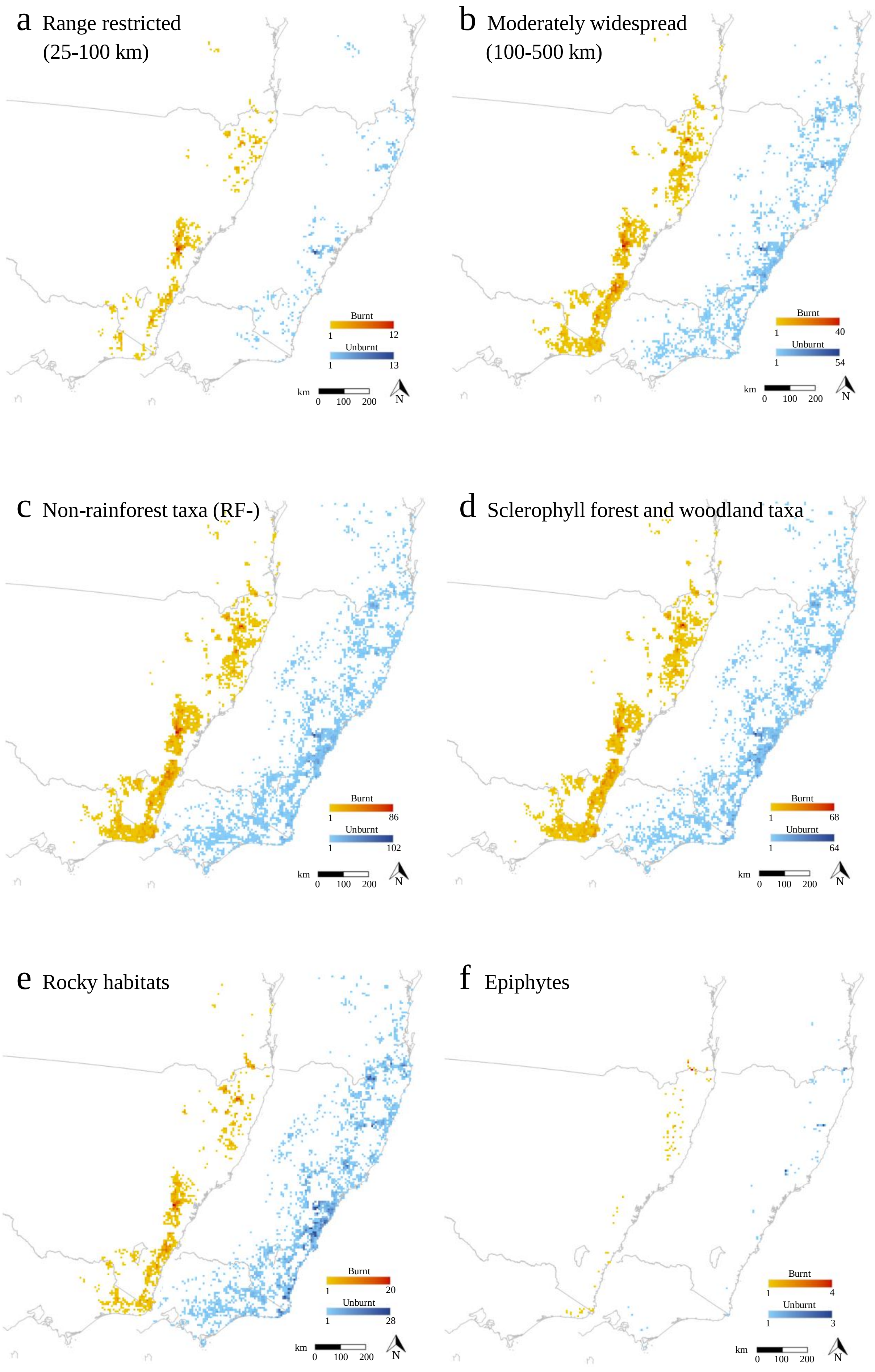

Supplementary Figure 3. Species richness based on burnt (red) and unburnt (blue) species location records aggregated to a 7.5 x 7.5 km grid. (a) Range restricted taxa with 25-100 km maximum range extent. (b) Moderately widespread taxa with 100-500 km maximum range extent. (c) Non-rainforest taxa. (d) Taxa found in sclerophyll forest and woodland. (e) Taxa often found in rocky habitats including exposed ridges, boulder fields, outcrops and cliffs. (f) Epiphytes.

Supplementary Table 1. Estimated area (ha) and percent of total (%) of broad vegetation types burnt during the 2019-2020 fires within 11 south-east Australian IBRA bioregions.

Bioregion(s)		Broad vegetation types (NVIS 5.1 Major Vegetation Group Codes below)[1]					
		Rainforests and Vine Thickets	Eucalypt Forests and Woodlands	Other Forests and Woodlands	Shrublands and Heathlands	Grasslands, Herblands, Sedgelands and Rushlands	Cleared, Non-Native Vegetation, Buildings
Name (CODE)	Fire metrics	1	2, 3, 4, 5, 11	6, 7, 8, 9, 10, 13, 15, 31	14, 16, 17, 18	19, 20, 21, 22	25
All bioregions	Fire area (ha)	328,901	7,337,880	248,963	217,740	59,124	2,059,123
	% burned	33.0	28.2	8.4	28.8	6.2	5.6
Coastal Bioregions (NET, NNC, SYB, SEC)	Fire area (ha)	244,645	4,397,004	100,845	174,589	12,423	824,961
	% burned	58.6	58.8	38.4	63.0	36.2	19.2
Australian Alps (AUA)	Fire area (ha)	34.0	426,732	1,139	3,490	21,941	3,995
	% burned	0.8	39.0	18.4	14.7	24.9	42.0
Brigalow Belt South (BBS)	Fire area (ha)	8,805	552,872	56,366	8,210	6,557	359,078
	% burned	5.6	6.8	3.0	3.4	2.0	2.2
Nandewar (NAN)	Fire area (ha)	1,421	104,666	33,623	1,823	1,073	49,059
	% burned	6.5	14.4	9.2	33.6	0.8	4.8
New England Tablelands (NET)	Fire area (ha)	13,058	467,003	41,737	11,949	5,307	234,635
	% burned	79.2	42.2	27.1	56.2	74.8	15.2
NSW North Coast (NNC)	Fire area (ha)	175,970	1,323,743	7,426	8,486	4,579	353,296
	% burned	59.6	59.4	31.7	30.0	32.5	27.0
NSW South Western Slopes (NSS)	Fire area (ha)	0	123,724	429	725	393	140,946
	% burned	0	11.6	0.3	1.1	0.3	2.1
South East Coastal Plain (SCP)	Fire area (ha)	0	1,522	0	20	0	5,039
	% burned	0	0.5	0	0.03	0	0.4
South East Corner (SEC)	Fire area (ha)	32,696	1,360,556	46,563	17,117	1,074	120,484
	% burned	82.8	67.1	72.3	42.2	24.2	36.0
South Eastern Highlands (SEH)	Fire area (ha)	10,158	1,070,714	8,592	14,771	5,594	261,803
	% burned	31.0	24.4	36.82	32.8	3.4	7.8
Southeast Queensland (SEQ)	Fire area (ha)	63,838	660,646	47,969	14,112	11,143	414,242
	% burned	17.6	23.8	20.8	34.6	19.5	10.0
Sydney Basin (SYB)	Fire area (ha)	22,921	1,245,702	5,119	137,037	1,463	116,546
	% burned	34.6	58.8	24.7	73.2	16.8	10.4

[1] Major Vegetation Groups by code are as follows: 1 = Rainforests and Vine Thickets, 2 = Eucalypt Tall Open Forests, 3 = Eucalypt Open Forests, 4 = Eucalypt Low Open Forests, 5 = Eucalypt Woodlands, 6 = Acacia Forests and Woodlands, 7 = Callitris Forests and Woodlands, 8 = Casuarina Forests and Woodlands, 9 = Melaleuca Forests and Woodlands, 10 = Other Forests and Woodlands, 11 = Eucalypt Open Woodlands, 13 = Acacia Open Woodlands, 14 = Mallee Woodlands and Shrublands, 15 = Low Closed Forests and Tall Closed Shrublands, 16 = Acacia Shrublands, 17 = Other Shrublands, 18 = Heathlands, 19 = Tussock Grasslands, 20 = Hummock Grasslands, 21 = Other Grasslands, Herblands, Sedgelands and Rushlands, 22 = Chenopod Shrublands, Samphire Shrublands and Forblands, 25 = Cleared, Non-Native Vegetation, Buildings, 31 = Other Open Woodlands.

Supplementary Table 2. Number of vascular plant taxa in different life forms by rainforest habitat category.

Life form		Rainforest Category				
Code	Name	RF-	RF	RF+	RF++	Total
AF	Annual forb	12				12
AG	Annual graminoid	2				2
AQ	Aquatic	3				3
CL	Climber	4	2	4	2	12
EP	Epiphyte			2	6	8
PF	Non-geophytic perennial forb	50	5	4	1	60
GF	Geophytic perennial forb	57	2			59
PG	Perennial graminoid	49	2	2	3	56
LS	Low shrub and subshrub	160	1	4		165
MS	Medium shrub	239	8	5	1	253
TS	Tall Shrub	64	11	3	4	82
LT	Low tree	37	4	4	7	52
MT	Medium tree	25		2	2	29
TT	Tall Tree	8	2		1	11
SFC	Short ferns and cycads	6	3		1	10
TFC	Tall ferns and cycads		1	1		2
Total		716	41	31	28	816

[1]RF++ = Rainforest, primarily or solely occurring in rainforests; RF+ = Semi-rainforest, occurring in rainforests but not restricted to them and frequently found in adjacent habitats, especially wet sclerophyll forests; RF = Rainforest margin, sometimes found around the margins of rainforest but more prevalent in other habitats and not typically considered a rainforest species; RF- = Non-rainforest, not considered a rainforest species and rarely if ever found in them.

Supplementary Table 3. Counts of species endemic to the study area classified by life form and range size class.

Life form		Maximum range extent[1]								
		ERR	VRR	RR	MWIDE (A)	MWIDE (B)	VWIDE (A)	VWIDE (B)	EWIDE	
Code	Name	0 to 10 km	10 to 25 km	25 to 100 km	100 to 250 km	250 to 500 km	500 to 750 km	750 to 1000 km	>1000 km	Total
LS	Low shrub and subshrub	6	8	36	49	35	6	5	3	148
MS	Medium shrub	22	20	52	61	40	17	12	12	236
TS	Tall Shrub	5	6	14	12	24	7	3	3	74
LT	Low tree	1	1	12	19	10	2	3	2	50
MT	Medium tree	1	1	5	6	6	3	2	0	24
TT	Tall Tree	1	0	1	2	3	2	1	0	10
GF	Geophytic perennial forb	5	5	12	17	8	2	1	1	51
PF	Non-geophytic perennial forb	4	5	5	10	7	4	1	6	42
CL	Climber	0	0	0	1	4	1	0	0	6
EP	Epiphyte	0	0	0	0	2	3	0	0	5
AF	Annual forb	2	0	2	1	1	1	2	1	10
AG	Annual graminoid	0	0	0	0	1	0	0	0	1
AQ	Aquatic	0	0	0	0	0	0	0	1	1
PG	Perennial graminoid	3	0	3	7	8	2	3	9	35
SFC	Short ferns and cycads	0	1	1	3	0	1	0	0	6
TFC	Tall ferns and cycads	0	1	0	0	0	0	0	0	1
Total		50	48	143	188	149	51	33	38	700

[1]ERR = extremely range-restricted, VRR = very range-restricted, RR = range-restricted, MWIDE (A) = moderately widespread, smaller; MWIDE (B) = moderately widespread, larger; VWIDE (A) = very widespread, smaller; VWIDE (B) = very widespread, smaller; EWIDE = extremely widespread.

Supplementary Notes

Supplementary Notes 1: Further details of range extent (RE_T) by habitat type (rainforest) category analysis.

This analysis was performed to compare the maximum range extent of species grouped by habitat type (i.e., rainforest, semi-rainforest, rainforest margin and non-rainforest). A boxplot containing these data is shown in Supplementary Notes Figure 1.

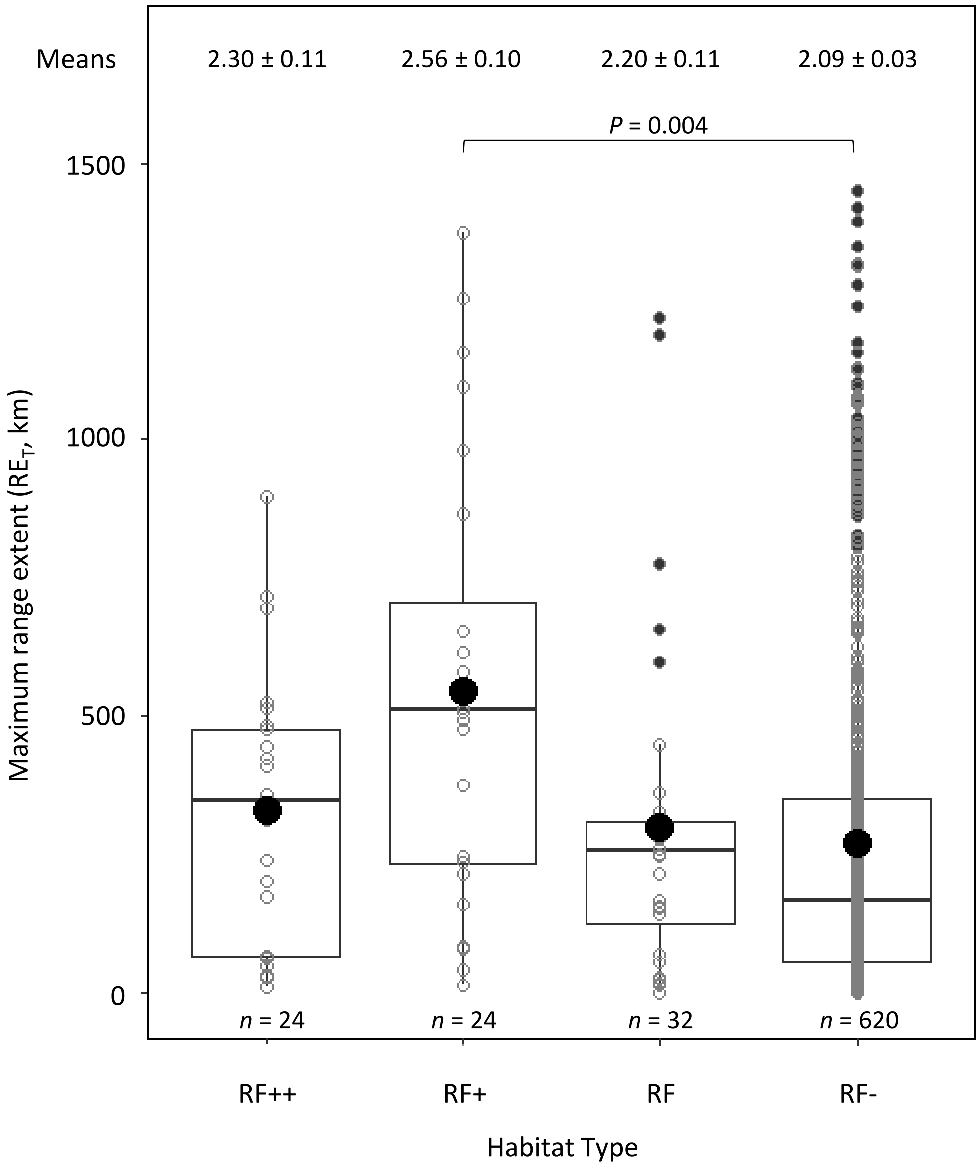

Supplementary Notes 1 Figure 1. Boxplot showing maximum range extent (RE_T) of plant species endemic to the south-east mainland study area grouped by habitat type category (RF++ = rainforest, RF+ = semi-rainforest, RF = rainforest margins, RF- = non-rainforests). Maximum range extent was determined as the maximum distance between any two specimen location records. Log-transformed means (± 1 SE) are shown at top, with significant (two-way tests at the 0.05 criterion) post-hoc means comparisons determined using Tukey's Honest Significant Difference method. Species numbers in each habitat type are provided below each group; data points are shown as small circles and group means (untransformed) are shown as large filled circles. The middle and lower and upper hinges of each boxplot correspond to the median and first and third quartiles respectively while whiskers extend to the largest value ≤1.5 times the interquartile range; data outliers are filled in black.

Supplementary Notes

Supplementary Notes 1: continued

One-way analysis of variance performed on log-transformed data indicate a significant difference among means: $F = 4.65$; df =3,696; $P = 0.003$. Post-hoc analysis indicates that semi-rainforest and non-rainforest species means were significantly different (two-way $P = 0.004$); all other mean differences were not significant at the 0.05 criterion.

Additional non-parametric tests yield similar results. Differences among habitat types were significant based on the Kruskal-Wallis rank sum test ($\chi^2 = 14.3$, df = 3, $P = 0.003$) and the RF+ vs. RF- comparison was significant based on two tailed pairwise Wilcox tests (P = 0.01). No other group differences were significant.

Supplementary Notes

Supplementary Notes 2: Further details of quantile regression of proportion burnt by range extent.

The relationship between maximum range extent (RE_T) and proportion of range or populations burnt (PF_M) was further investigated because it showed a strongly triangular relationship. The results of quantile regression show a steep relationship between maximum range extent (RE_T) and proportion burnt (PF_M) at the 0.90 quantile ($PF_M = 1.006 – 0.00033RE_T$; $P < 0.001$), with PF_M falling by roughly 0.15 (15% of range burnt) per 500 km increase in range (Supplementary Notes Figure 2). The number of species with very large proportions of their ranges burnt declines with range size, with very few species that have ranges over 750 km experiencing fire over 75% or more of their range.

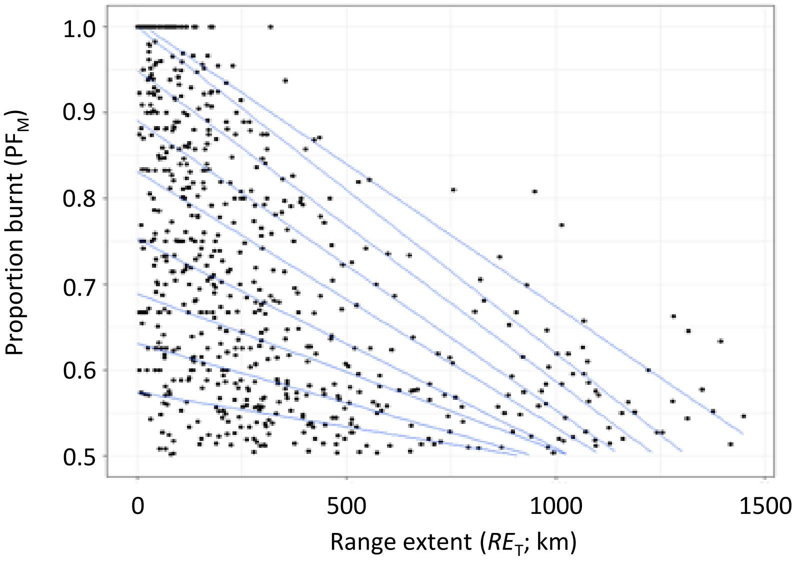

Supplementary Notes 2 Figure 2. Results of quantile regression, showing linear regression models for quantiles 0.10, 0.20….0.90.

The estimated slope coefficient is also significantly less steep than that of the least square estimate (red line; dashed = 90% confidence interval) at low quantiles (0.10-0.40) and steeper at higher quantiles (0.60 – 0.80).

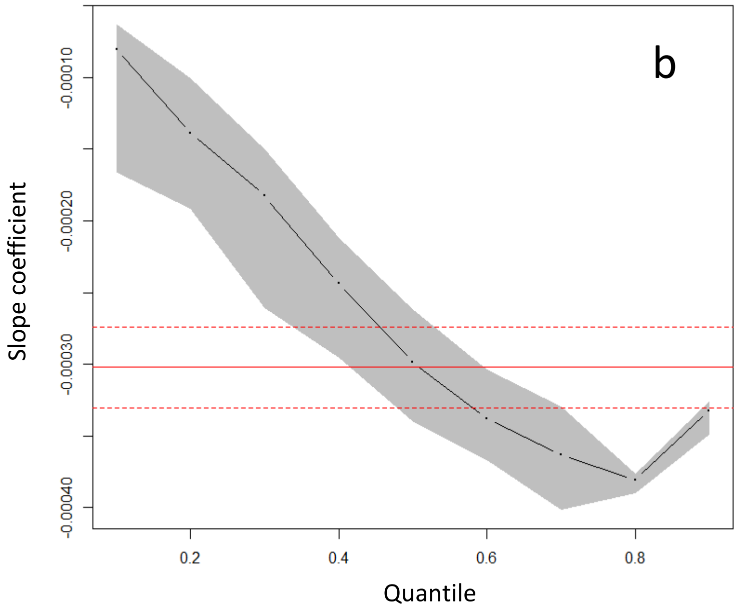

Supplementary Notes 2 Figure 2. Results of quantile regression, showing changes in slope coefficient across quantiles. The 90% confidence interval is shaded in gray.

Supplementary Notes

Supplementary Notes 3
Contingency analysis of life form by range extent

The aim of this analysis was to investigate whether any relationship existed between range extent (RE_T) and life form across all species endemic to the study region. The primary analysis of this relationship was provided in Figure 3c and the accompanying legend text. We also conducted a contingency analysis of the same relationship but with grouped RE_T and life form categories. Both categories were grouped because many cell values in ungrouped data contained counts of less than 5 (Supplementary Table 3), particularly for life form classes containing few species.
The contingency table was as follows:

Table 1. Counts of species in six life form classes across four range extent classes. E+VRR = extremely to very range restricted, maximum range extent (RE_T) < 25km; RR = range restricted, RE_T = 25 to <100 km; MWIDE = moderately widespread, RE_T = 100 to <500 km), V+EWIDE = very to extremely widespread, RE_T > 500 km.

	Range extent class				
Life form class	E+VRR	RR	MWIDE	V+EWIDE	Total
Low shrubs	14	36	84	14	148
Medium shrubs	42	52	101	41	236
Tall shrubs	11	14	36	13	74
Trees	5	18	46	15	84
Perennial Forbs	19	17	42	15	93
Other	7	6	28	24	65
Total	98	143	337	122	700

Contingency analysis was performed using the R base package ‘stats’ version 4.0.1. The result indicated the presence of a highly significant row-column relationship (Pearson χ^2 = 42.1, df = 15, P = 0.0002). Pearson residuals are as follows:

Table 2. Pearson residuals

	Range extent class			
Life form group	E+VRR	RR	MWIDE	V+EWIDE
Low shrubs	-1.5	1.0	1.5	-2.3
Medium shrubs	1.6	0.5	-1.2	0.0
Tall shrubs	0.2	-0.3	0.1	0.0
Trees	-2.0	0.2	0.9	0.1
Perennial Forbs	1.7	-0.5	-0.4	-0.3
Other	-0.7	-2.0	-0.6	3.8

Supplementary Notes

Supplementary Notes 4
List of R packages used

R version 4.0.1 (2020-06-06)
Platform: x86_64-w64-mingw32/x64 (64-bit)
Running under: Windows Server x64 (build 14393)

Matrix products: default

Attached base packages:

grid, tools, stats, graphics, grDevices, utils, datasets, methods, base

Other attached packages:

quantreg_5.75, SparseM_1.78, Cairo_1.5-12.2, ggspatial_1.1.4, ggthemes_4.2.0, extrafontdb_1.0, extrafont_0.17, gridExtra_2.3, mapview_2.9.0, dismo_1.3-3, rJava_0.9-13, forcats_0.5.0, purrr_0.3.4, readr_1.4.0, tidyr_1.1.2, tibble_3.0.4, ggplot2_3.3.2, tidyverse_1.3.0, stringr_1.4.0, dplyr_1.0.2, smoothr_0.1.2, stars_0.4-3, abind_1.4-5, sf_0.9-6, rgeos_0.5-5, raster_3.4-5, rgdal_1.5-18, sp_1.4-4, moments_0.14

MaxEnt version 3.4.1[1] (available at https://biodiversityinformatics.amnh.org/open_source/maxent/) was used for species distribution modelling.

[1]Steven J. Phillips, Miroslav Dudík, Robert E. Schapire. [Internet] Maxent software for modeling species niches and distributions (Version 3.4.1). Available from url: http://biodiversityinformatics.amnh.org/open_source/maxent/. Accessed on 2020-09-19.
