# Supplementary material for: Implications of the 2019–2020 megafires for the biogeography and conservation of Australian vegetation
Source: Nat Commun. 2021 Feb 15;12:1023. doi: 10.1038/s41467-021-21266-5 (PMC7884386; doi:10.1038/s41467-021-21266-5)
Supplement: Supplementary file 3 — Description of Additional Supplementary Information [file 41467_2021_21266_MOESM3_ESM.pdf]

**Description of Additional Supplementary Files**

**File Name**: Supplementary Data 1

**Description:** Some major bush fires events across south-eastern Australia, 1850-2016.

**File Name:** Supplementary Data 2

**Description:** List of species with more than 50% of geocoded location records or predicted ranges occurring within the south-east Australian mainland fires, including model, range, life form, trait and habitat data.

**File Name**: Supplementary Data 3

**Description:** Fire trait data for 270 taxa with more than 50% of geocoded location records or predicted ranges occurring within the south- east Australian mainland fires.
