# Supplementary material for: Implications of the 2019–2020 megafires for the biogeography and conservation of Australian vegetation
Source: Nat Commun. 2021 Feb 15;12:1023. doi: 10.1038/s41467-021-21266-5 (PMC7884386; doi:10.1038/s41467-021-21266-5)
Supplement: Supplementary file 7 — Reporting summary [file 41467_2021_21266_MOESM7_ESM.pdf]

nature research

Corresponding author(s): Robert Godfree

Last updated by author(s): 10/01/2021

Reporting Summary

Nature Research wishes to improve the reproducibility of the work that we publish. This form provides structure for consistency and transparency in reporting. For further information on Nature Research policies, see our Editorial Policies and the Editorial Policy Checklist.

Statistics

For all statistical analyses, confirm that the following items are present in the figure legend, table legend, main text, or Methods section.

n/a	Confirmed	
☐	☒	The exact sample size (n) for each experimental group/condition, given as a discrete number and unit of measurement
☒	☐	A statement on whether measurements were taken from distinct samples or whether the same sample was measured repeatedly
☐	☒	The statistical test(s) used AND whether they are one- or two-sided<br>*Only common tests should be described solely by name; describe more complex techniques in the Methods section.*
☒	☐	A description of all covariates tested
☐	☒	A description of any assumptions or corrections, such as tests of normality and adjustment for multiple comparisons
☐	☒	A full description of the statistical parameters including central tendency (e.g. means) or other basic estimates (e.g. regression coefficient) AND variation (e.g. standard deviation) or associated estimates of uncertainty (e.g. confidence intervals)
☐	☒	For null hypothesis testing, the test statistic (e.g. F, t, r) with confidence intervals, effect sizes, degrees of freedom and P value noted<br>*Give P values as exact values whenever suitable.*
☒	☐	For Bayesian analysis, information on the choice of priors and Markov chain Monte Carlo settings
☒	☐	For hierarchical and complex designs, identification of the appropriate level for tests and full reporting of outcomes
☒	☐	Estimates of effect sizes (e.g. Cohen's d, Pearson's r), indicating how they were calculated

Our web collection on statistics for biologists contains articles on many of the points above.

Software and code

Policy information about availability of computer code

Data collection

All raw data was freely available and downloaded from the following websites using the Firefox web browser Version 78.6.0:

(1) Fire hotspot data: Geoscience Australia - Digital Earth Australia website (https://hotspots.dea.ga.gov.au/), (2) Interim Biogeographic Regionalisation for Australia (IBRA), Version 7: https://www.environment.gov.au/land/nrs/science/ibra; (3) Present Major Vegetation Groups - NVIS Version 5.1 at http://www.environment.gov.au/land/native-vegetation/national-vegetation-information-system/data-products; (4) AVH Specimen Data: www.ala.org.au (Atlas of Living Australia); (5) Environmental layers: CSIRO Data Access Portal (https://data.csiro.au)

Data analysis

Data analysis was performed using the following software:

(1) AVH specimen dataset cleaning: openrefine version 3.3 [58b839b] and R version 4.0.1 (2020-02-29) run in R Studio Version 1.2.5033
(2) Statistical analyses: R stats base package version 4.0.1; quantreg version 5.75; ggplot2 Version 3.3.2; moments Version 0.14
(3) Modelling: raster version 3.4-5; dismo version 1.3-3; rJava version 0.9-13, MaxEnt version 3.4.1

A detailed list of all software used is provided in the Supplementary Notes. All R code used in the study is publicly available on the CSIRO Data Access Portal (https://doi.org/10.25919/sd7h-ff33).

For manuscripts utilizing custom algorithms or software that are central to the research but not yet described in published literature, software must be made available to editors and reviewers. We strongly encourage code deposition in a community repository (e.g. GitHub). See the Nature Research guidelines for submitting code & software for further information.

Data

Policy information about availability of data

All manuscripts must include a data availability statement. This statement should provide the following information, where applicable:

- Accession codes, unique identifiers, or web links for publicly available datasets
- A list of figures that have associated raw data
- A description of any restrictions on data availability

Source data and output data that support the results reported in this study are publicly available on the CSIRO Data Access Portal (https://data.csiro.au) at https://doi.org/10.25919/sd7h-ff33. This includes fire hotspot, NVIS and IBRA source datasets downloaded under Creative Commons Attribution 4.0 International Licence and derived fire layers. Data used to generate statistics reported in the paper are available in Supplementary Data 2.

Field-specific reporting

Please select the one below that is the best fit for your research. If you are not sure, read the appropriate sections before making your selection.

☐ Life sciences ☐ Behavioural & social sciences ☒ Ecological, evolutionary & environmental sciences

For a reference copy of the document with all sections, see nature.com/documents/nr-reporting-summary-flat.pdf

Ecological, evolutionary & environmental sciences study design

All studies must disclose on these points even when the disclosure is negative.

Study description	This study investigates the impacts of the 2019-2020 fires on plant species and communities within the south-eastern Australian study region. It involved analysis of pre-existing datasets or new datasets that we developed from online and textual sources. We used hotspot satellite data to generate fire maps which were then used to quantify fire extent and temperature. Vegetation and bioregion metadata were used to model and map Australian vegetation and quantify fire impacts in different Australian bioregions. Herbarium specimen data was required to quantify plant species ranges within the study area and to determine the impact of the fires at the species level. Trait and habitat data were then required to understand the potential impacts of the fires on different kinds of plant species.
Research sample	1. Historic hotspot data: downloaded from Geoscience Australia - Digital Earth Australia website (https://hotspots.dea.ga.gov.au/)<br>2. Plant species location data for south-eastern Australia from the Australasian Virtual Herbarium were downloaded from the Atlas of Living Australia on January 24, 2020 (https://doi.org/10.26197/5e2bcb71d290c).<br>3. The Australian bioregion map defined in the IBRA (Interim Biogeographic Regionalisation for Australia) Version 7 (2012) was downloaded from https://www.environment.gov.au/fed/catalog/search/resource/details.page?uuid=%7B4A2321F0-DD57-454E-BE34-6FD4BDE64703%7D<br>4. The Major Vegetation Groups map defined under the National Vegetation Information System (NVIS) Version 5.1 were download from http://environment.gov.au/fed/catalog/search/resource/details.page?uuid=%7B991C36C0-3FEA-4469-8C30-BB56CC2C7772%7D.<br>5. Fire response data were sourced primarily from the New South Wales Flora Fire Response Database (supplied by Fire Ecology Unit, NSW Office of Environment and Heritage, Hurstville, NSW 1481)<br>6. Environmental layers used in Maxent modelling (9s resolution data) are available from the CSIRO Data Access Portal at https://doi.org/10.25919/5dce30cad79a8 (Bio4-Bio32), https://doi.org/10.4225/08/5afa9f7d1a552 (EAA, EPI, WDI and WDX) and https://doi.org/10.4225/08/5b285fd14991f (CLY, SND, NTO, PTO, BDW and TWI3S).<br>7. Species life form and habitat data were determined using data provided in the Atlas of Living Australia (ALA; www.ala.org.au), NSW Flora Online (https://plantnet.rbgsyd.nsw.gov.au/), VICFLORA
Sampling strategy	The study initially involved use of satellite hotspot, IBRA and NVIS datasets and herbarium specimen records to generate fire maps and to quantify the impact of the fires on Australian plant communities. Trait data, fire response data and species distribution modelling were used to assess the possible responses of different groups of species to the fires. These were required to extend the scope of the paper to address reviewer and editorial comments.
Data collection	Source data used in the study were downloaded from freely available sources and software described in the Supplementary Notes were used to analyse and generate data. Species life form, habitat data, fire response data and fire history available in Supplementary Data 1, Supplementary Data 2 and Supplementary Data 3 were collated from data available on the Atlas of Living Australia (ALA; www.ala.org.au), NSW Flora Online (https://plantnet.rbgsyd.nsw.gov.au/), VICFLORA, the New South Wales Flora Fire Response Database, searchable online databases and expert elicitation.
Timing and spatial scale	Timing: July 1 2019 - 11 February 2020 (bushfires under investigation)<br>Spatial scale: south mainland Australia (bounding 144.01, -39.17; 154.0, -25.341)
Data exclusions	Herbarium specimen data, which initially included plant and fungal taxa, were cleaned as part of the original submission to remove unreliable records as described in detail in the Methods section. Data then further cleaned and filtered by removing older records

	and non-vascular plant taxa to address reviewer concerns over data quality. Satellite hotspot data were filtered to remove spurious records and improve accuracy of fire maps as described in the Methods. Code is available on the CSIRO Data Portal. We designed the strategy and code to perform all data cleaning and exclusion.
Reproducibility	The data are not experimental; all results can be reconstructed from Supplementary Data and data and code has been deposited online and made publically available
Randomization	This is a descriptive, non-experimental study and used comprehensive pre-existing satellite hotspot, vegetation community and plant species data. Randomised background points were generated and used in Maxent species distribution modelling. Fire response data was collated for plant species as available.
Blinding	No experimental treatments that would require blinding were applied in any way as part of this study.

Did the study involve field work? ☐ Yes ☒ No

Reporting for specific materials, systems and methods

We require information from authors about some types of materials, experimental systems and methods used in many studies. Here, indicate whether each material, system or method listed is relevant to your study. If you are not sure if a list item applies to your research, read the appropriate section before selecting a response.

Materials & experimental systems

n/a	Involved in the study	
☒	☐	Antibodies
☒	☐	Eukaryotic cell lines
☒	☐	Palaeontology and archaeology
☒	☐	Animals and other organisms
☒	☐	Human research participants
☒	☐	Clinical data
☒	☐	Dual use research of concern

Methods

n/a	Involved in the study	
☒	☐	ChIP-seq
☒	☐	Flow cytometry
☒	☐	MRI-based neuroimaging
